# Scintillation of polyester fabric and clothing via proton irradiation and its utilization in surface imaging of proton pencil beams

**DOI:** 10.1038/s41598-024-62456-7

**Published:** 2024-06-12

**Authors:** Seiichi Yamamoto, Tomohiro Yamashita, Masao Yoshino, Kei Kamada, Akira Yoshikawa, Teiji Nishio, Jun Kataoka

**Affiliations:** 1 Faculty of Science and Engineering, Waseda University, Tokyo, Japan; 2 Kobe Proton Center, Kobe, Japan; 3 New Industry Creation Hatchery Center (NICHe), Tohoku University, Sendai, Japan; 4 Division of Health Sciences, Graduate School of Medicine, Osaka University, Osaka, Japan

**Keywords:** Imaging and sensing, Imaging techniques, Experimental nuclear physics

## Abstract

In the realm of radiation therapy, a conspicuous obstacle lies in the dearth of external observation concerning radiation beams aimed at the patient. While real-time monitoring of such beams on the patient's surface during therapy holds promise, the imaging of particle beams has thus far proven to be a formidable task. Here, we show our discovery of polyester fabrics and cloths as auspicious scintillating materials, ideally suited for the visualization of radiation beams upon the patient's surface. The light output of polyester fabrics ranged from 10 to 20% of that observed in plastic scintillators. When exposed to spot scanning proton beams, clear beam spots emerged on the surface of the polyester cloths. The movement of these scanning beams was effectively captured using a CMOS camera in a light-shield-free with lights-off environment. The resulting images provided a means for evaluating spills of the proton beams. The inherent flexibility of polyester fabrics and clothing enhances their appeal for applications in the intricate landscape of radiation therapy, promising a bright future for surface beam imaging endeavors.

## Introduction

The outcome of radiotherapy relies on the precision of radiation dose delivery to the target tumor. However, due to factors such as patient movement, anatomical variations, and uncertainties in treatment delivery parameters, incorrect doses may be administered, posing a potential risk of patient injury^1^. The ability to adapt flexibly to changes in patient position during treatment, monitoring patient position and radiation beam without additional radiation exposure, is an optimal approach for providing high-quality treatments.

Cherenkov imaging serves as a real-time verification method for patient treatment^2–5^. When charged particles move through a medium at a speed exceeding that of light, Cherenkov light can be emitted. This technique captures light emitted from tissues during the phenomenon known as the Cherenkov-light emission. Cherenkov imaging systems visually provide the treatment team with the radiation irradiated field on the patient's surface^6^, allowing real-time confirmation of treatment delivery or storage for post-treatment review. Cherenkov imaging in X-ray from LINAC has been suggested to detect multi-leaf collimator (MLC) shapes and movements, identify patient misalignments affecting accurate treatment delivery, and improve incident detection in radiation quality assurance programs^7^.

However, in particle beam therapy, such as proton therapy, Cherenkov light is rarely generated with the proton beam energy used for treatment^8^, making real-time imaging challenging. Although water and biological tissues emit light for radiation with energy below the Cherenkov light threshold^8–10^, the emitted light is minimal,requiring a dark box and a highly sensitive camera as well as long time is required for observation^8–10^.

To address this challenge, we propose scintillating fabric and clothing; that is subjects in therapy were considered to wear body fit clothing that emits light by the irradiation of radiation, potentially allowing us to monitor proton beams during irradiation. Upon investigating potential scintillating materials, we discovered that polyester fabric and clothing could serve as promising candidates capable of emitting light when exposed to radiation. This observation was partly motivated by the finding that polyethylene terephthalate (PET) plates exhibit scintillation upon irradiation^11^, with both materials having the same chemical formula ((C_10_H_8_O_4_)_n_). This report elucidates that polyester fabrics and clothing such as T-shirt or caps indeed emit light when exposed to radiation, scrutinizes their light-emitting characteristics, and shows their utility in proton beam spot monitoring through irradiation experiments.

## Results

### Measurement of energy spectra of polyester fabrics for alpha particles

**Figure 1:**
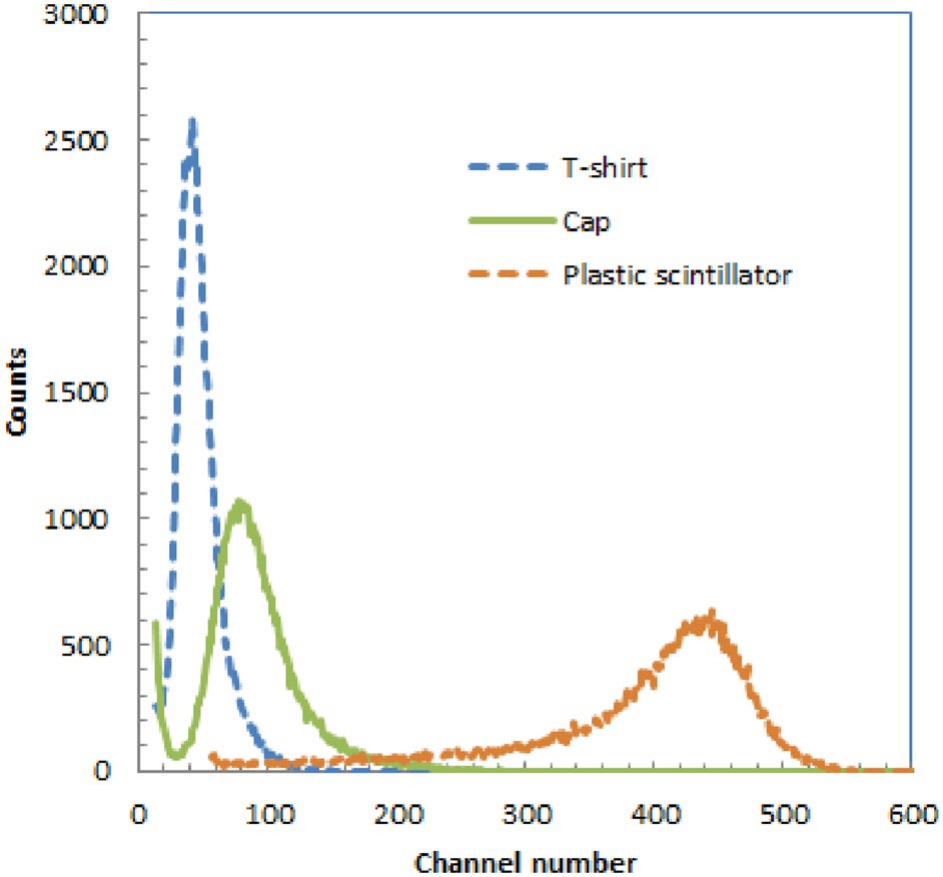
Energy spectra of T-shirt, swimcap andplastic scintillator for 5.4 MeV alpha particles.

In Fig. 1, we show the energy spectra of the polyester T-shirt and cap alongside that of a plastic scintillator for 5.4 MeV alpha particles. The horizontal axis depicts the intensity of light produced in the material due to alpha particle irradiation, while the vertical axis shows the count of events related to this light production. Therefore, the peak channel number directly reflects the quantity of light emitted by the material as a result of alpha particle irradiation. While the energy peaks were modest for both the T-shirt and cap, each showed a distinct single peak in their respective spectra. Notably, the energy spectrum for the cotton T-shirt did not yield any signals, only generating noise signals from the photomultiplier tube (PMT). Table 1 provides a summary of the relative peak channels for the T-shirt and cap in comparison to the plastic scintillator. Absolute light output was calculated by assuming the light production of plastic scintillator was 1000 photons /MeV for alpha particles.

**Table 1:** Relative peak channels and absolute light output for T-shirt and cap compared with plastic scintillator for alpha particles.

	Plastic scintillator	Polyester T-shirt	Polyester cap
Relative peak channel	100	9.6	18
Absolute light output (photons /MeV)	1000	96	180

### Measurement of X-ray-induced radio-luminescence spectra

**Figure 2:**
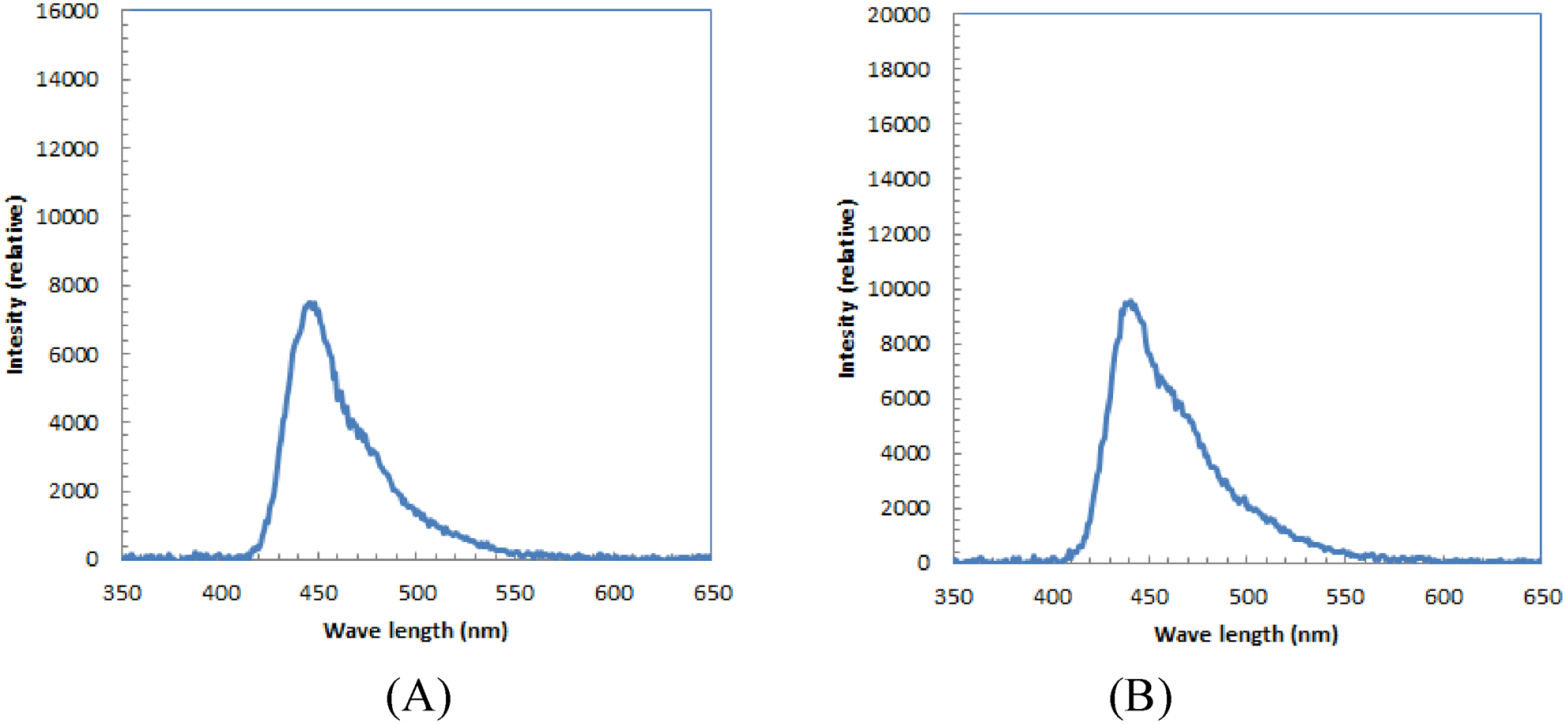
Measured radio-luminescence spectra of polyester T-shirt (**A**) and swim cap (**B**) by the X-ray irradiation.

The measured radio-luminescence spectra of the scintillating clothing and caps, obtained through X-ray irradiation, are depicted in Fig. 2. Both spectra exhibited a range from approximately 400–600 nm, with a distinct and similar sharp peak occurring at around 440 nm.

### Imaging of polyester T-shirt and cap during irradiation of proton beams

While proton beams were being irradiated, clear beam spots were observed on both the polyester T-shirt and cap, whereas the cotton T-shirt did not produce any observable signal.

**Figure 3:**
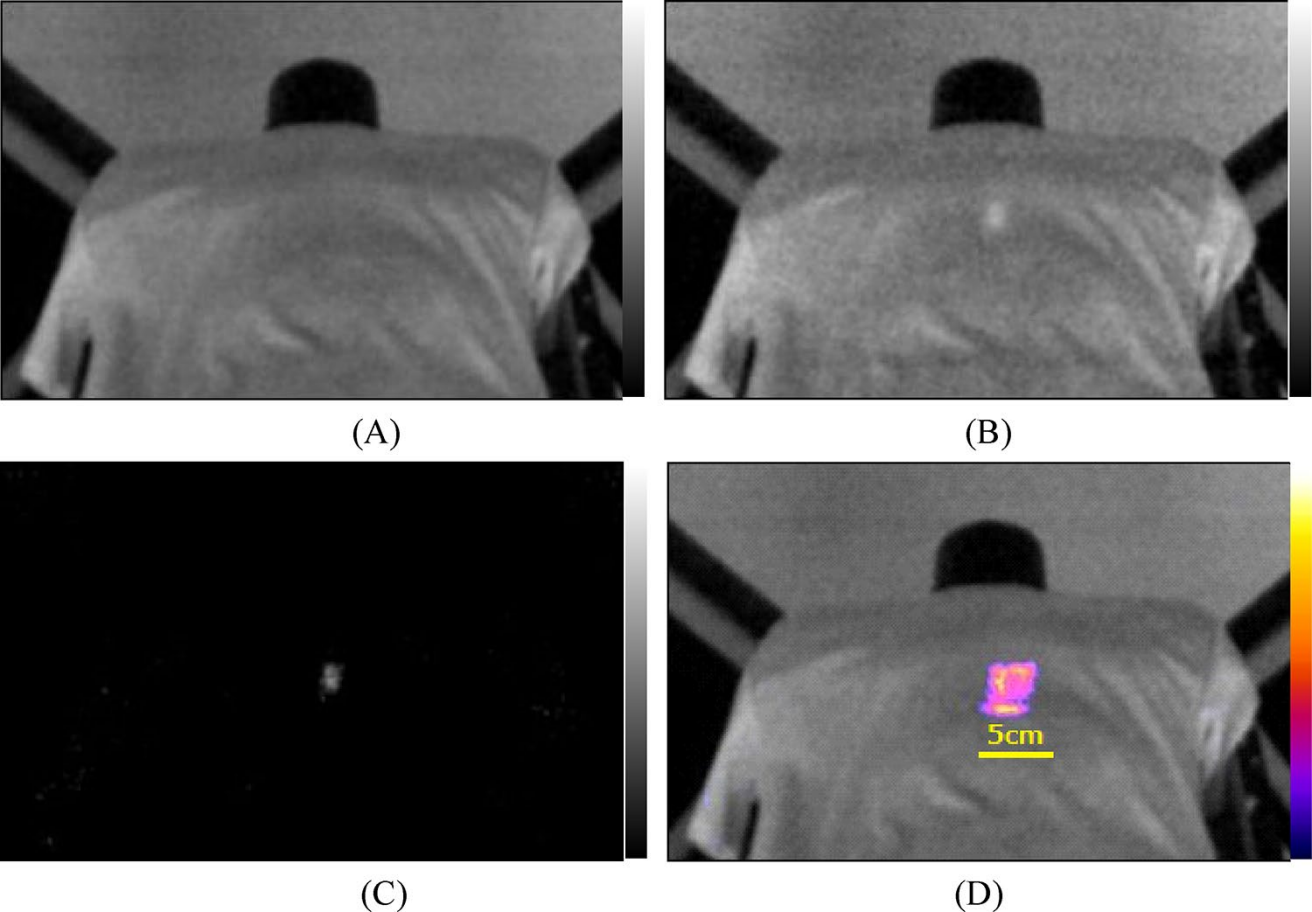
Images of polyester T-shirt during irradiation of proton beams: off-beam image (**A**), on-beam image (**B**), background subtracted image (**C**) and fused image of accumulated (color area) and off-beam images (**D**).

In Fig. 3A, the off-beam image of the polyester T-shirt during proton beam irradiation is presented. No scintillating spots were observed because the proton beam was not irradiated to the polyester T-shirt at this time frame between the spills. Moving to Fig. 3B, the on-beam image of the polyester clothing during proton beam irradiation is shown. A clearly visible scintillating spot at the center of the image is observed in the center of T-shirt during the irradiation of the spot scanning proton beam.

Figure 3C shows the subtracted image of the polyester clothing during proton beam irradiation. In this image, only the scintillating spot is visible, resulting from the irradiation of the spot scanning proton beam. Finally, Fig. 3D shows the fused image of the 75 frame accumulated subtracted image and off-bema image of the polyester T-shirt during proton beam irradiation. The accumulated beam irradiated area is distinctly visible in color, with the off-beam image in black and white. We show a video of beam images of polyester T-shirt combined with accumulated images arranged side by side during irradiation of proton beam in Supplemental material 1.

**Figure 4:**
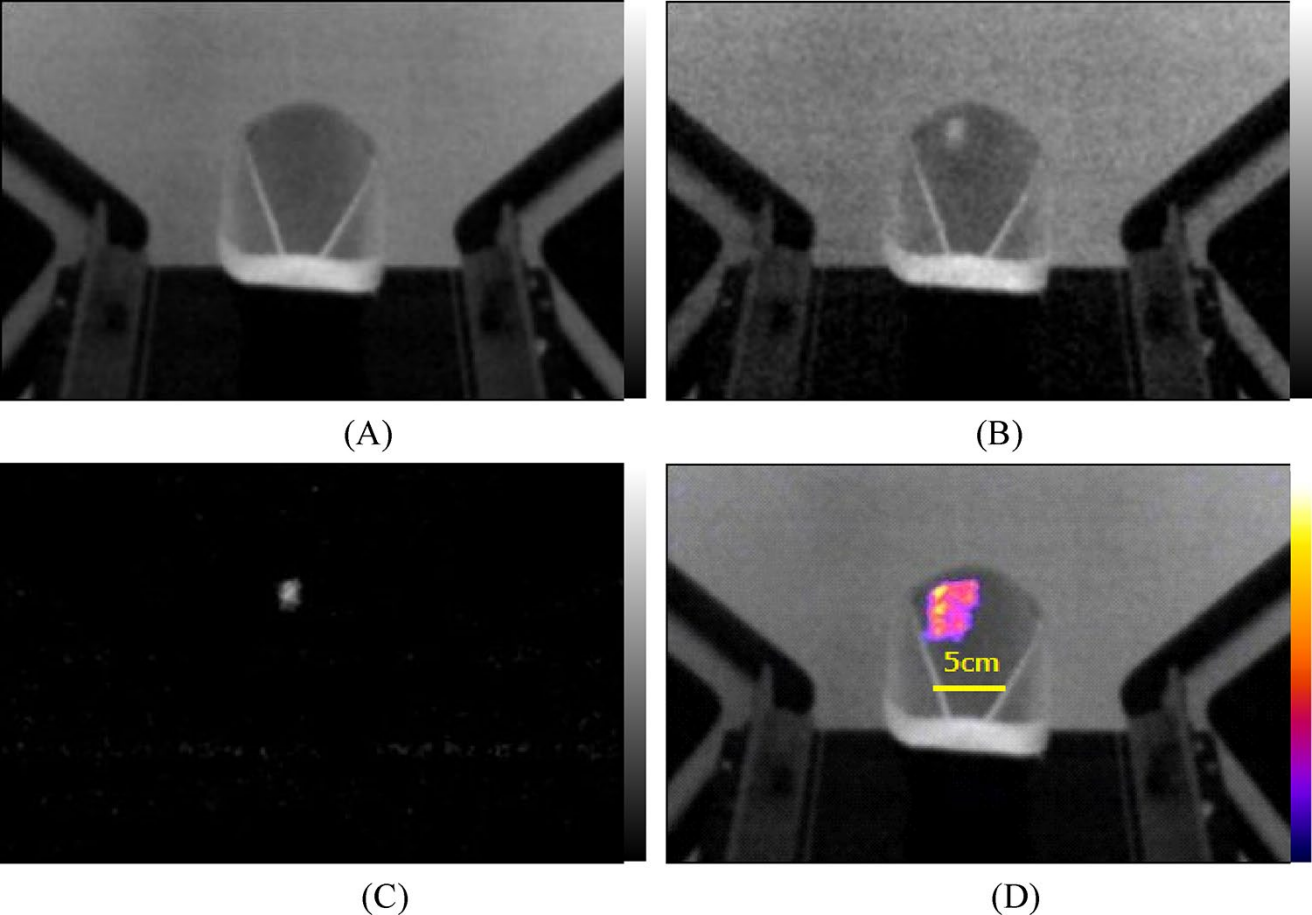
Images of polyester cap during irradiation of proton beams: off-beam image (**A**), on-beam image (**B**), background subtracted image (**C**) and fused image of accumulated (color area) and off-beam images (**D**).

Figure 4A shows the off-beam image of polyester swim cap during irradiation of proton beams. We could not observe any scintillating spot because the proton beam was not irradiated to the polyester cap at this time frame. We show the on-beam image of polyester cap during irradiation of proton beams in Fig. 4B. We could clearly observe the scintillating spot at the left upper part of the cap by the irradiation of the spot scanning proton beam.

Figure 4C shows the subtracted image of polyester cap during irradiation of proton beams. In this image, only the scintillating spot is visible, resulting from the irradiation of the spot scanning proton beam. We show the fused image of the 75 frame accumulated image and off-bema image of polyester cap during irradiation of proton beams in Fig. 4D. We could clearly observe the beam irradiated area at the left upper part of the cap in color by the irradiation of the spot scanning proton beam with the off-beam image in black and white. We show a video of beam images of polyester cap combined with accumulated images arranged side by side during irradiation of proton beam in Supplemental material 2.

**Figure 5:**
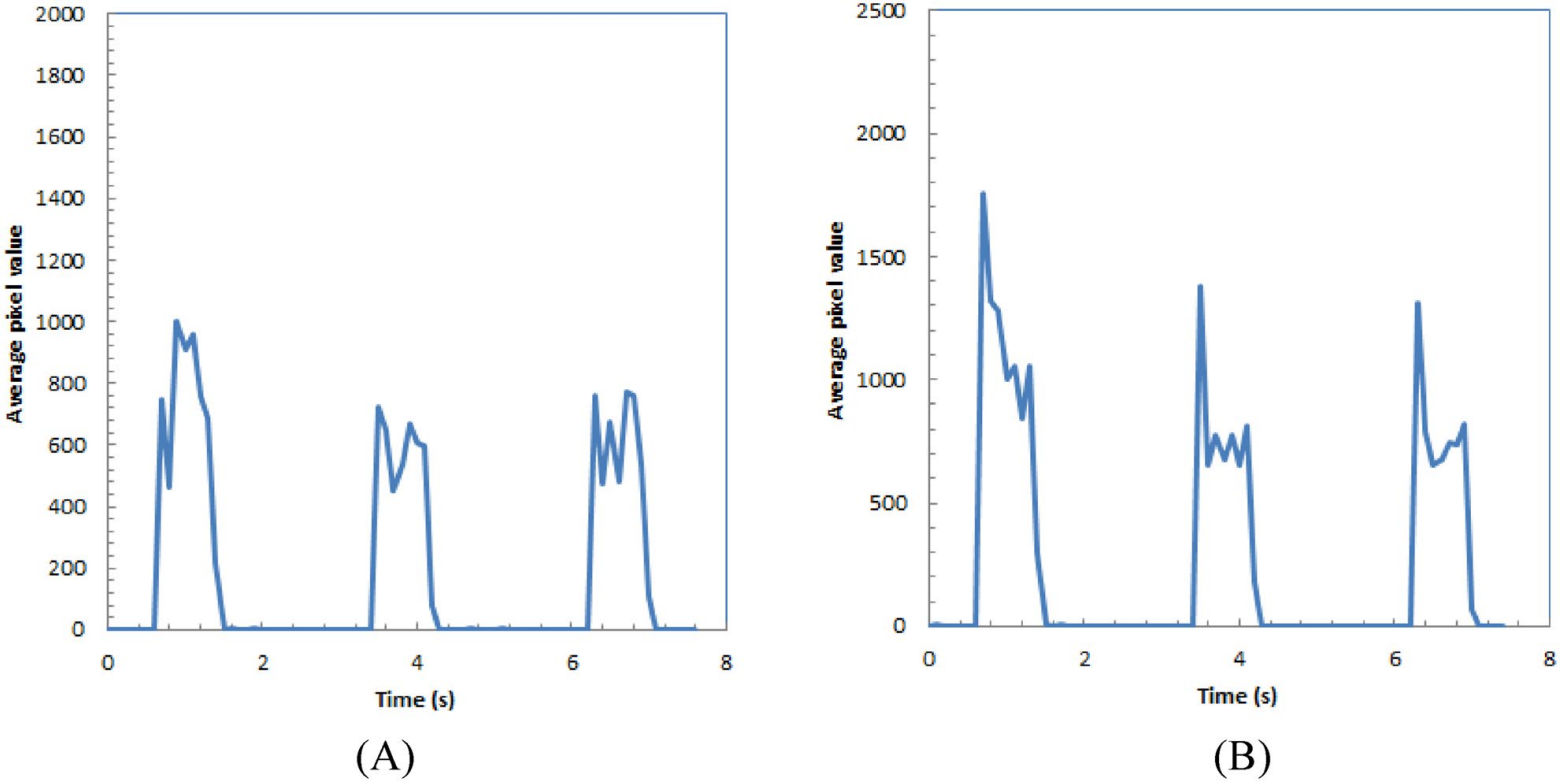
Time intensity curve of spot part in subtracted images of polyester T-shirt (**A**) and cap (**B**) during irradiation of proton beams.

In Fig. 5A,B, the time intensity curves for the spot regions in the subtracted images of the polyester T-shirt and cap during proton beam irradiation are shown, respectively. Both curves exhibit peaks with approximately 1-s durations, followed by bottoms lasting around 2 s, and these variations occur at a frequency of 3.8 Hz, roughly matched to the spill duration of the proton therapy system (3.6 Hz).

The images of plastic scintillator plate showed more than 10 times brighter intensity spot during irradiation of proton beams. We summarize the relative intensity of the spots in the images in Table 1 for the polyester T-shirt and cap compared with that of the plastic scintillator measured by the CMOS camera when the proton beams were irradiated.

## Discussions

We successfully imaged clear beam spots on the surface of the polyester T-shirt and cap. The movement of these scanning beams was effectively recorded using a CMOS camera in a light-shield-free environment with the lights off. Since the beam spots surface imaging was possible with the almost clinical dose level (5 Gy) and clinically used spot scanning condition, imaging of patient in therapy will be possible. Although we selected the polyester T-shirt and the swim cap this time for the imaging, other polyester based fabric and clothing will also emit light by the irradiation of radiations and applicable to spot scanning proton beam surface imaging.

One advantage of real-time surface beam imaging during proton therapy is the potential detection of beam errors associated with the proton therapy system although it will be rare, such as scanning beam errors. Without real-time surface imaging, we may not detect beam errors related to these hardware issues in the proton therapy system. Confirmation of the visible beam spots on the patient surface by our proposed imaging system will give the confidences for irradiated positions of the proton beams on the patient surface as well as the rough intensity changes of proton beams irradiated to the patient.

The relative light production of the polyester fabrics in the T-shirt and cap, as indicated by the PMT-measured data in Table 1, was approximately 10% to 20% of that of the plastic scintillator. Meanwhile, the light production measured by the CMOS camera images for these materials, as listed in Table 2, was around 6% of that of the plastic scintillator. The observed differences in light production between the PMT and CMOS camera measurements can be attributed to the nature of the beams; the PMT data were obtained with 5.4 MeV alpha particles, whereas the CMOS camera data were obtained with proton beams with energies around 130.6 MeV. Since the 5.4 MeV alpha particles were absorbed in the polyester fabrics (0.2–0.3 mm thick) and plastic scintillator plate (1 mm thick), the energy spectra exhibited those with the full energy of alpha particles. Conversely, as proton beams were higher in energy, only a portion of the energy was absorbed in the fabrics or plastic scintillator plate. The thicker plastic scintillator (1 mm) emitted more light, resulting in relatively smaller light production for polyester fabrics with protons compared to alpha particles.

**Table 2:** Relative intensity of beam in image for T-shirt and cap compared with plastic scintillator measured by CMOS camera when proton beams were irradiated.

plastic scintillator	Polyester T-shirt	Polyester cap
100	5.9	6.7

Proton beam spots on the polyester T-shirt and cap were observable with the CMOS camera in a light-shield-free environment, but this required exposure times longer than 0.1 s. For imaging proton beams with shorter exposure times, a higher sensitivity camera or scintillating clothing with greater light production will be necessary. Also in the case of surface measurement in patients, challenges arise from the significant dependence on the installation positions and the number of observation cameras. This includes concerns about whether it is possible to visualize the irradiation distribution on the complex surface of the body.

The radio-luminescence spectra revealed that the scintillating wavelength of the T-shirt and caps was shorter than the maximum sensitivity of the CMOS camera, leading to a decrease in the camera image intensity. Utilizing scintillating clothing with longer wavelengths that align with the sensitivity of the CMOS sensor may provide an additional advantage of imaging with shorter imaging time or imaging with a higher background light level in the treatment room during patient proton therapy.

In an application for FLASH proton therapy^12,13^, although many of the irradiation techniques proposed for FLASH proton therapy at present are passive irradiation method, the amount of emitted light measured is substantial, making it advantageous for distribution measurements with high contrast and short time.

The suggested polyester fabrics and clothing for surface imaging in radiation therapy are not limited to proton therapy; they are also suitable for other forms of radiation therapy, including carbon-ion, high-energy X-rays, and electrons from LINAC. Our next objectives involve conducting imaging on patients undergoing proton therapy and exploring trials for radiation therapy beams other than proton beams. This is aimed at broadening the applications of the proposed materials and methods, extending beyond proton therapy.

We have shown that polyester fabrics and clothing emit detectable light when exposed to spot-scanning proton beams in a light-shield-free environment, captured by a CMOS camera. The versatility and flexibility of polyester fabrics and clothing make them highly valuable for surface beam imaging during proton therapy. In conclusion, polyester fabrics and clothing show promise for efficient surface beam imaging during proton therapy, and we anticipate their clinical application in surface beam monitoring.

## Materials and methods

### Exploring potential scintillating fabric and clothing

Initially, we investigated potential materials that could serve as scintillating fabrics and clothing, identifying several reports on materials that exhibit scintillation upon exposure to radiation. Darafsheh et al. discovered that polymethyl methacrylate (PMMA) emits scintillation light at approximately 415 nm when exposed to proton irradiation^14^. They also found that bare silica optical fibers emit light during proton irradiation^15^.

The light emission from PMMA or silica optical fibers under proton irradiation was identified not as Cherenkov light but as scintillation, indicating their potential as scintillating materials. However, given that the light emission from these materials is about 10 photons/MeV for particle ions^16–18^, their application in radiation therapy would require a high dose rate and a completely dark room. While float glass emits a higher amount of light (~ 250 photons/MeV) upon irradiation by particle ions^16,19,20^ and could be used for beam imaging, fabricating fabric and clothing from it poses significant challenges.

Alternatively, reports have documented that polyethylene terephthalate (PET) plates demonstrate scintillation upon radiation exposure^11^. We noticed that the chemical similarity between polyester and polyethylene terephthalate (PET), which share the molecular formula ((C_10_H_8_O_4_)_n_), and we identified polyester fabric and clothing as promising materials for scintillation applications. Accordingly, we measured the light output from polyester when exposed to radiation and conducted imaging during such exposure.

### Polyester fabric and clothing used for the measurements

We sourced polyester fabrics and clothing from various manufacturers to identify the best candidates for imaging experiments, conducting preliminary screenings by evaluating their light emission in response to alpha particles. Although all tested polyester fabrics and clothing showed scintillation with alpha particle irradiation, we specifically selected a T-shirt and a swim cap to further assess their scintillation characteristics and imaging capabilities. These items were chosen based on their optimal thickness, the absence of any additional coatings on their surfaces, and their suitability for scintillation imaging of thoraco-abdominal regions and the heads of patients undergoing proton therapy.

**Figure 6:**
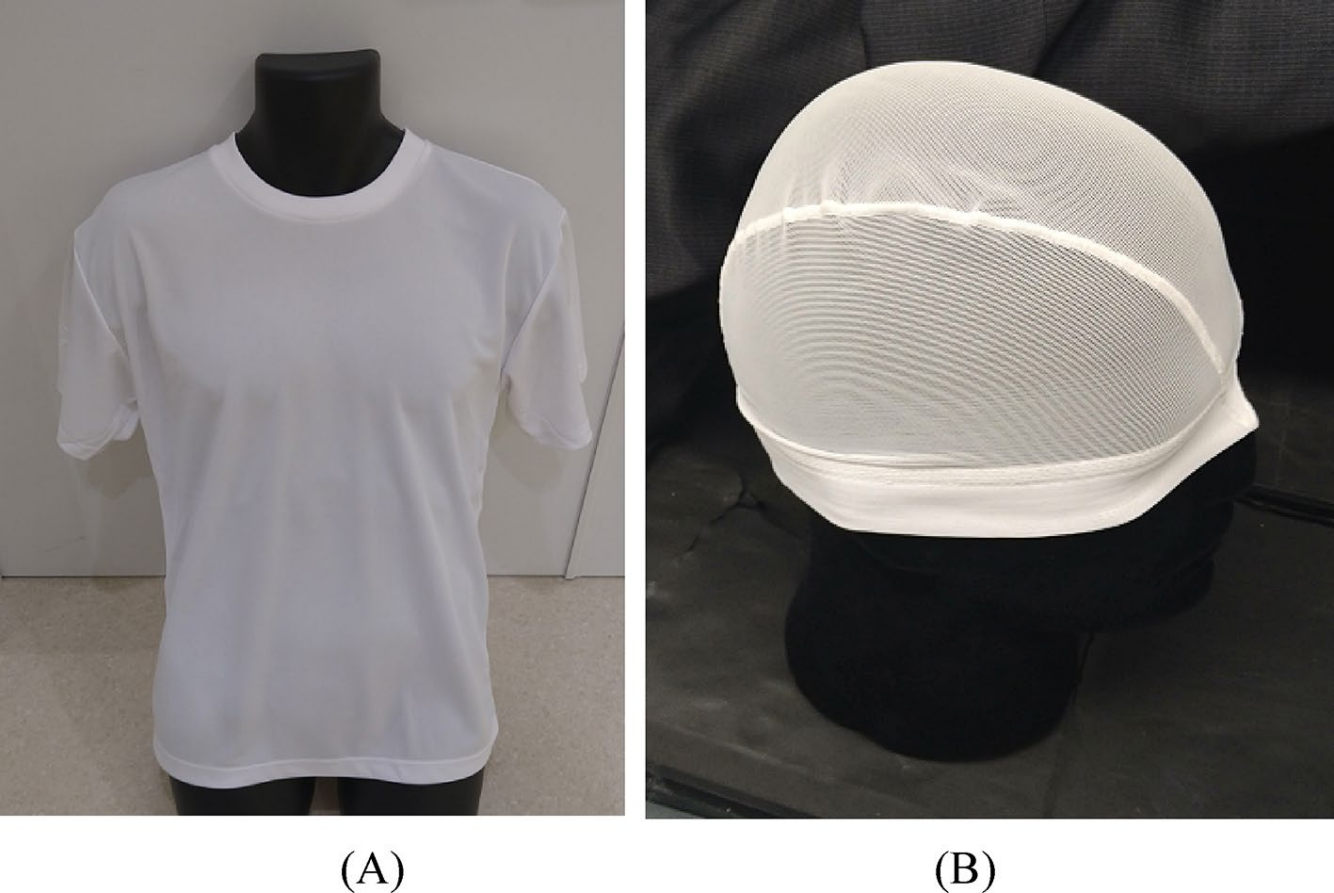
Photos of polyester T-shirt (**A**) and swim cap (**B**) used for measurements.

In Fig. 6A,B, we show photos of the polyester T-shirt and cap, respectively. The T-shirt used for measurements was a commercial product (Glimmer, Japan), with a 100% polyester composition and a white color. The swim cap used for the experiment was also a commercial product (Footmark, Japan), with a composition of 80% polyester and 20% polyurethane, both in white. The thickness of the polyester fabric in the T-shirt and cap was approximately 0.2 mm and 0.3 mm, respectively. The polyester fabric in the cap featured numerous small rectangular openings (approximately 0.3 mm × 0.5 mm) probably designed for water drainage. The cost of each item was less than 10 US dollars. Additionally, we included a white cotton T-shirt (Print star, Japan) for confirming that the produced scintillation by the irradiation of alpha particles was below the detection limit.

### Measurement of energy spectra for alpha particles

To quantify the light emission from the polyester T-shirt and cap in response to radiation, we assessed their performance by exposing them to alpha particles. The light output of these scintillating materials is a crucial parameter for capturing scintillation images by optical camera of therapy beams with high contrast and short exposure times.

**Figure 7:**
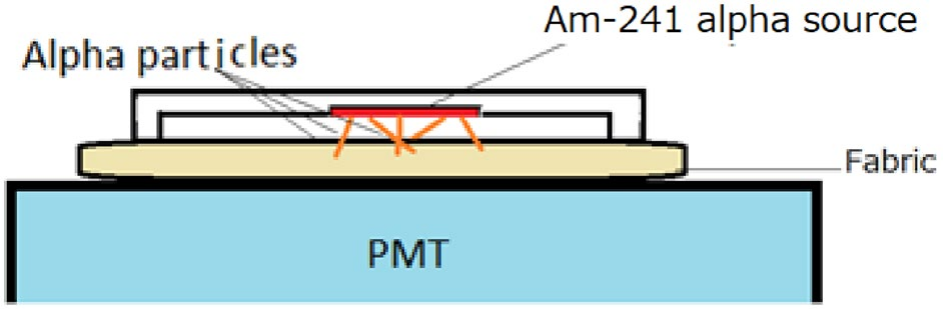
Schematic drawing of energy spectra measurement of polyester clothing and cap.

For these measurements of energy spectra for alpha particles, a sample of the clothing (~ 2 cm × 2 cm and 0.2 mm for T shirt and 0.3 mm for cap) cut from the edges was positioned atop a PMT (Hamamatsu Photonics, R6233-100HA). Alpha particles from an Am-241 alpha source were directed towards the upper surface of the materials, as shown in Fig. 7. Since the thickness of the samples was larger than the range of alpha particles in the materials (less than 0.1 mm). The Am-241 source used in these measurements was an uncoated one with a radioactivity of 2 kBq, emitting alpha particles at an energy of 5.4 MeV.

The energy spectra of the tested clothing for alpha particles were compared with those obtained from a ~ 2 cm × 2 cm, 1 mm thick plastic scintillator plate (Eljen Technology, USA). This comparison allowed us to evaluate the relative light output of the polyester T-shirt and cap in response to alpha particles.

### Measurement of X-ray-induced radio-luminescence spectra

The X-ray induced radio-luminescence spectra of the scintillating clothing and caps are crucial factors in selecting an optical camera for capturing scintillation images of therapy beams with high sensitivity. To analyze the emission spectra of the polyester clothing and cap in response to radiation, X-ray-induced radio-luminescence was measured. Measurements were made by using a spectrometer (Shamrock 163, Oxford Instruments, a compact Czerny-Turner spectrograph, combined with an iDus420-OE, Oxford Instruments, an open-electrode charge-coupled device). The X-ray tube was operated at a tube voltage of 40 kV and a tube current of 25 mA. The X-ray exposure time was 100 s. Scintillation from a piece of polyester clothing or cap was transmitted to the spectrometer through a quartz optical fiber. The measured radioluminescence spectra were corrected for the spectrometer's sensitivity spectrum as provided by the manufacturer.

### Imaging of polyester T-shirt and cap during irradiation of proton beams

**Figure 8:**
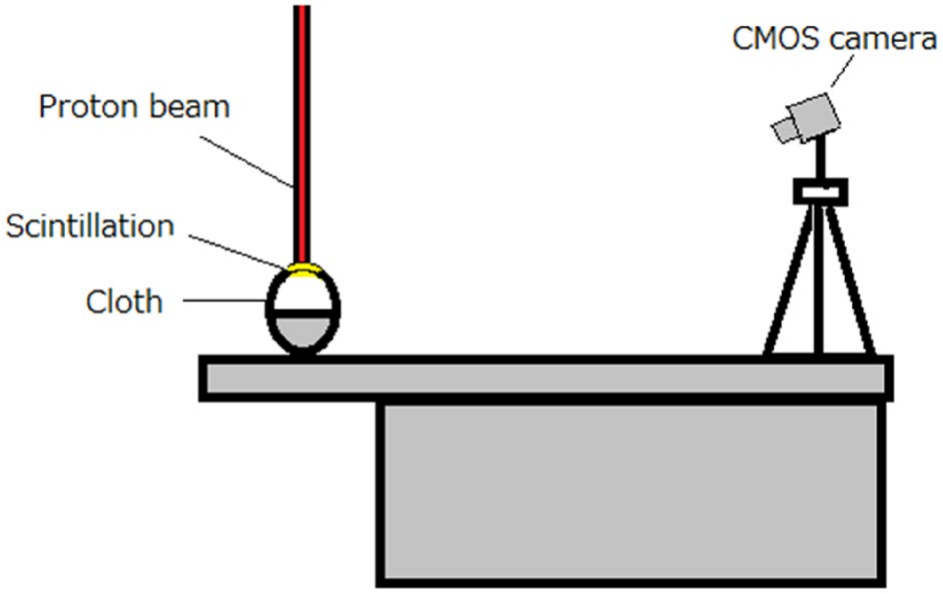
Schematic drawing of the imaging of polyester T-shirt and cap during irradiation of proton beams.

As shown in Fig. 8, the polyester T-shirt or cap was positioned on the clinically utilized bed of the spot scanning proton therapy system (Hitachi, Japan) at Kobe Proton Center. The polyester T-shirt was placed on a black plastic manikin to replicate the body surface's shape, while the polyester cap was mounted on a black head-shaped Styrofoam to maintain its stretched form.

The proton therapy system, based on a synchrotron, had a spill rate of 0.36 Hz and a maximum energy of 235 MeV. We positioned a CMOS camera (Bitran, CS-700 M, Japan) attached a C-mount F-1.4 lens (Computar, Japan) at a horizontal distance of ~ 2 m and a vertical distance of ~ 1 m from the polyester T-shirt or cap. The possible imaging wavelength of the CMOS camera was 400 nm to 1000 nm, and the maximum sensitivity wavelength was ~ 600 nm. This setup allowed us to capture images of the proton beam scintillation generated on the polyester clothing or cap from the upper front side. When the imaging, we did not use the dark box but we turned off all the lighting fixtures in the room and covered two large panel displays by black cloths in the treatment room to reduce the environmental light. There remained some small environmental lights in the room such from LEDs.

We captured images of the proton beam with a spread-out Bragg peak (SOBP) created using spot scanning beams with dimensions of 5 cm × 5 cm × 5 cm and a range of 12 cm. The maximum proton energy of the SOBP beam was 130.6 MeV. The SOBP beam was chosen for its efficiency in observing the movement of the beam spot on the subject. The beam intensity matched the clinically used one, delivering a dose of 5 Gy. Each imaging session lasted for 10 s, measuring 100 continuous frame images with a 95 ms exposure time and a 100 ms interval, thus the imaging was conducted at only a part of the SOBP beam irradiation with the energy of the proton beam (~ 130.6 MeV) with the field of view of ~ 5 cm (vertical direction) ×  ~ 3 cm (horizontal direction).

### Image processing, production of background subtracted images, accumulated images and videos

The images recorded by the CMOS camera were processed by a software application (ImageJ). Measured 100 images were stacked and several neighbor images with the beam spots observed (on-beam) and non-observed (off-beam) were added to create higher signal to noise ratio (SNR) images. These on-beam and off-beam images were also used to calculate the background subtracted images by subtracting the off-beam image from on-beam-image.

Similar procedures were conducted by subtracting the summed off-beam image from the stack image to create the background subtracted stack images. The background subtracted images were summed for 100 images to obtain an accumulated background subtracted image after baseline noise in each background subtracted image was removed. Also fused images were formed by adding the background subtracted accumulated image and off-beam image with different color to clearly show the accumulated beam spots. In order to synchronize the starting time of the proton beam between T-shirt and cap imaging, a continuous sequence of 75 frames was utilized for image processing.

From the background subtracted stack images, time intensity curves were derived to check whether the spill intervals could be evaluated from the images. Also all the stack images including the subtracted stack images were converted to videos to show the actual movement of the spot scanning beams during irradiation of SOBP beams.

### Supplementary Information

Supplementary Legends.

Supplementary Video 1.

Supplementary Video 2.

## Data Availability

The datasets used and/or analyzed during the current study available from the corresponding author on reasonable request.
