# Supplementary material for: Scintillation of polyester fabric and clothing via proton irradiation and its utilization in surface imaging of proton pencil beams
Source: Sci Rep. 2024 Jun 12;14:13494. doi: 10.1038/s41598-024-62456-7 (PMC11169216; doi:10.1038/s41598-024-62456-7)
Supplement: Supplementary file 1 — Supplementary Legends. [file 41598_2024_62456_MOESM1_ESM.docx]

Legends of Supplemental material

Supplemental material-1

Video of beam images (left) and accumulated images (right) of polyester T-shirt during irradiation of proton beam.

Supplemental material-2

Video of beam images (left) and accumulated images (right) of polyester cap during irradiation of proton beam.
